# Duodeno-Jejunal Varicosities Following Extrahepatic Portal Vein Thrombosis

**DOI:** 10.1155/1992/16894

**Published:** 1992

**Authors:** Nick Varsamidakis, Brian R. Davidson, Kenneth Hobbs

**Affiliations:** Hepatobiliary and Liver Transplantation Unit University Department of Surgery Royal Free Hospital Pond Street London NW3 2QG UK

## Abstract

A 31 year old man, under investigation for melena, was found at endoscopy to have varicosities at the site of a duodeno-jejunostomy which had been performed for duodenal atresia when he was three days old. Angiography revealed an occluded portal vein with an extensive collateral circulation. At laparotomy some of the collateral vessels were found to pass through the anastomotic site and directly into the left lobe of the liver. The portal pressure was found to be minimally elevated. Resection of the anastomotic segment was performed with reconstruction using a Roux en Y jejunal loop. Bleeding from collateral vessels passing through an anastomosis site in a patient with extrahepatic portal vein thrombosis has not previously been reported.


*HPB Surgery*, 1992, Vol. 5, pp. 147–153
Reprints available directly from the publisher
Photocopying permitted by license only

© Harwood Academic Publishers GmbH
Printed in the United Kingdom

### CASE REPORT

### DUODENO-JEJUNAL VARICOSITIES FOLLOWING EXTRAHEPATIC PORTAL VEIN THROMBOSIS

NICK VARSAMIDAKIS, BRIAN R. DAVIDSON and KENNETH HOBBS
*Hepatobiliary and Liver Transplantation Unit, Royal Free Hospital, Pond Street, London NW3 2QG*

*(Received 1 November 1991)*

A 31 year old man, under investigation for melena, was found at endoscopy to have varicosities at the site of a duodeno-jejunostomy which had been performed for duodenal atresia when he was three days old. Angiography revealed an occluded portal vein with an extensive collateral circulation. At laparotomy some of the collateral vessels were found to pass through the anastomotic site and directly into the left lobe of the liver. The portal pressure was found to be minimally elevated. Resection of the anastomotic segment was performed with reconstruction using a Roux en Y jejunal loop. Bleeding from collateral vessels passing through an anastomosis site in a patient with extrahepatic portal vein thrombosis has not previously been reported.

KEY WORDS: Portal vein thrombosis, duodenal atresia, venous collaterals, varices

### INTRODUCTION

Thrombosis of the extrahepatic portal vein results in the development of large collateral veins which continue the portal perfusion of the liver (hepatopetal flow)[1]. Patients with this disorder usually maintain normal liver function but are at risk of gastro-intestinal bleeding from these abnormal vessels. The oesophagus and stomach are the most common sites although others have rarely been reported[2,3,4].

A patient is presented in whom upper gastro-intestinal haemorrhage occurred from collateral portal vessels at the site of a duodeno-jejunal anastomosis. Such a case has not previously been described.

### CASE REPORT

A 31 year old man was transferred to the Hepatobiliary Unit at the Royal Free Hospital for investigation of recurrent episodes of melena.

In his past medical history he was born with duodenal atresia and underwent a duodeno-jejunostomy at the age of three days. Post-operatively he required

Address correspondence to: Mr. B.R. Davidson, University Department of Surgery, Royal Free Hospital Medical School, Pond Street, London NW3 2QG

umbilical vein catheterisation for monitoring and had a prolonged ileus. At the age of two years he had an episode of melena and another one year later. Following this he remained well for 17 years when at the age of 20 he presented with a major upper gastrointestinal haemorrhage. On endoscopy bleeding varicosities were found at the site of the duodeno-jejunal anastomosis. He had no signs of liver disease, normal liver function tests and a normal liver biopsy. This episode of bleeding settled with conservative treatment as did further episodes of melena 2, 4, 5 and 8 years later. At the age of 29 he had a massive upper gastro-intestinal bleed from the site of his anastomosis and was referred to another hospital for surgery. Despite a mesocaval H graft being performed he continued to bleed and had two further episodes of melena requiring hospital admission and blood transfusion.

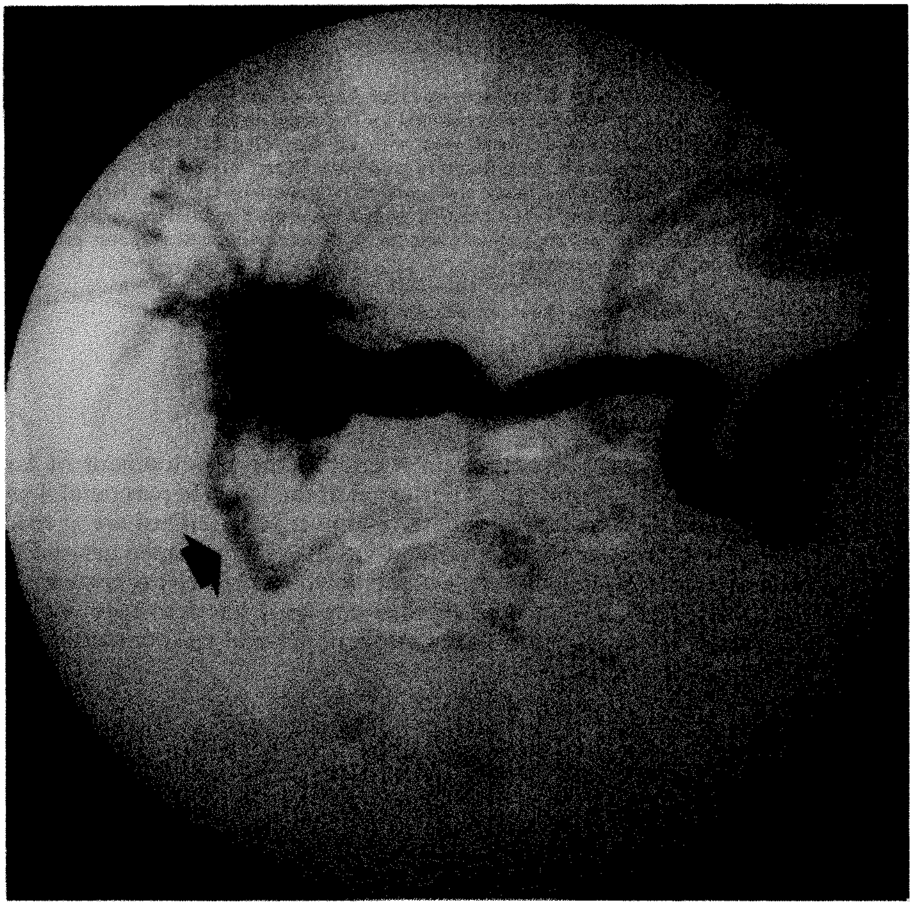

**Figure 1** Angiography (Splenoportography). On venography large collateral vessels are present at the hilus of the liver and there is no evidence of portal vein filling. A vessel is seen which corresponds to that visualised at the anastomotic site on endoscopy (arrowed).

On admission to the Royal Free Hospital he appeared pale but had a normal pulse rate and blood pressure. There were no signs of liver disease. Haemoglobin level was 3g/dl (normal 13-16g/dl). His clotting status was normal. Upper gastrointestinal endoscopy was carried out by an expert in injection sclerotherapy for varices and a normal oesophagus and stomach was found. At the site of his duodeno-jejunostomy, however, there were several friable and bleeding varicosities. Visceral angiography, including splenic portography (Figure 1), showed occlusion of the portal vein with massive collateral vessels at the hilum of the liver and at the bleeding site visualised at endoscopy. Flow through the collateral vessels was hepatopetal. There was no flow of contrast through the mesocaval shunt and the splenic pulp pressure was only minimally elevated at 13cm of water. Abdominal CT scanning with oral contrast showed his distal stomach and the site of his duodeno-jejunostomy to be closely adherent to the left lobe of the liver (Figures 2a and 2b). When intravenous contrast was administered the bridge of tissue between the distal stomach/duodenum and the left lobe of the liver was enhanced suggesting that it was vascular or well vascularised. As a result of these investigations further surgery was carried out.

The abdomen was opened through a right subcostal incision and dense vascular adhesions were encountered. The site of the duodeno-jejunostomy was found to be densely adherent to the left lobe of the liver. Within the small bowel mesentery and in the hepatorenal pouch were grossly distended veins running towards the hilum of the liver. Some of the veins within the small bowel mesentery coursed through the adherent anastomosis and entered the left lobe of the liver directly. The pressure within the enlarged mesenteric veins was 15cm of water. The anastomosis was mobilised from the liver with ligation of the traversing vessels and the site of the duodeno-jejunostomy divided. This allowed the involved segment of jejunum to be resected. Intestinal continuity was restored with a Roux en Y jejunal loop.

Histology of the resected specimen showed a length of small bowel and a small segment of duodenum. Abnormally large veins were confirmed within the mucosa and submucosa of the small bowel. At the site of the previous anastomosis there was loss of the mucosa overlying the varicosities.

Post operatively he developed a fistula at the site of his duodeno-jejunostomy which closed spontaneously with conservative treatment. Six months post operatively he has had no further gastrointestinal bleeding.

### DISCUSSION

In the case described major gastrointestinal haemorrhage was occurring from varicosities at the site of a duodeno-jejunostomy. In view of the findings of extrahepatic portal vein thrombosis and large collateral venous channels throughout the rest of the small bowel mesentery it seems likely that these vessels were also large hepatopetal venous collaterals passing through the site of the anastomosis into the liver. Their development at this site may be explained by dense adherence of the duodeno-jejunal anastomosis to the liver substance in the neonatal period following the surgery for duodenal atresia.

Several possibilities exist as to the aetiology of the extrahepatic portal vein thrombosis which is likely to have occurred at an early age in view of bleeding episodes starting at two years of age. The most likely cause would seem to be

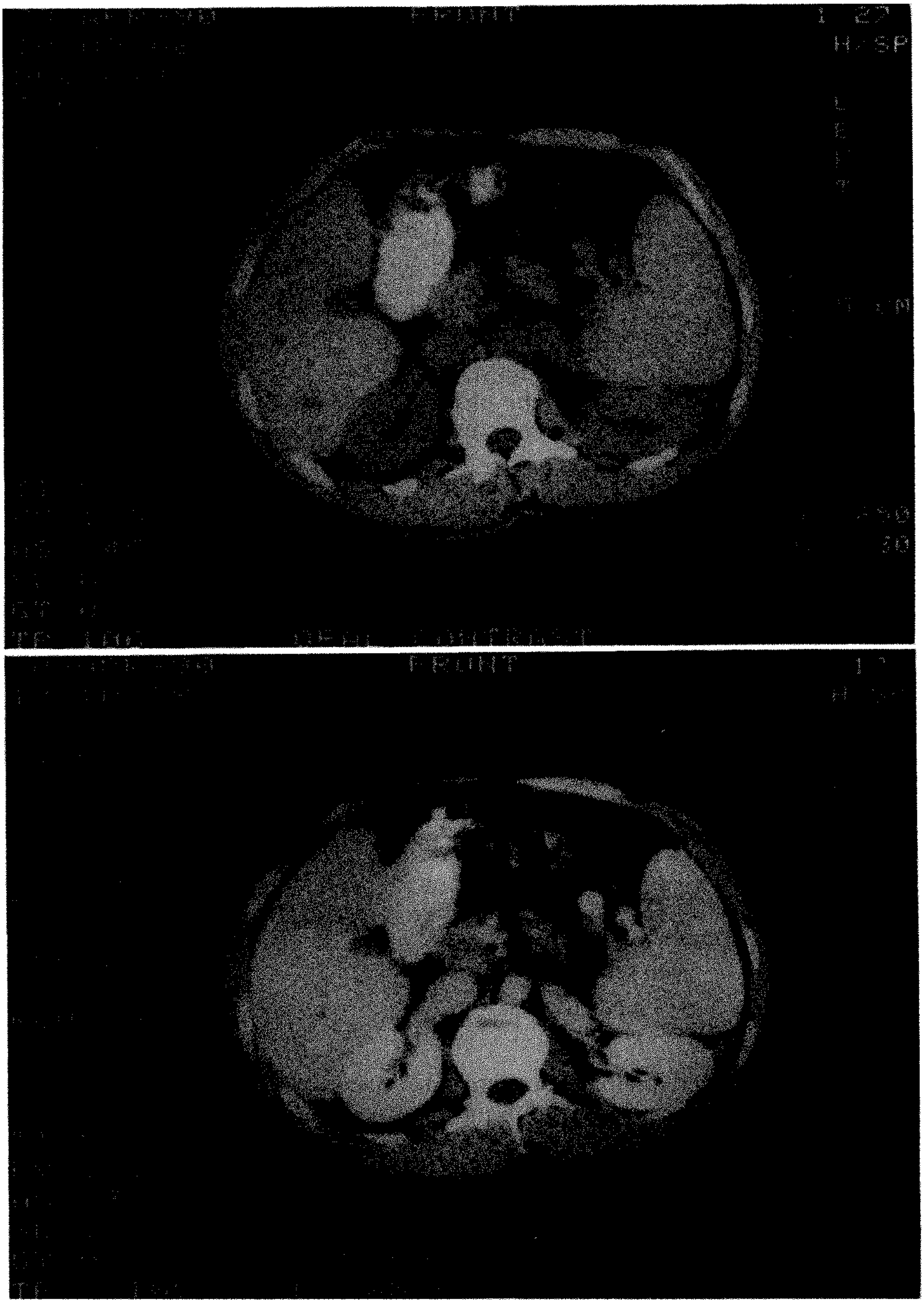

**Figure 2** (a and b) Abdominal CT Scanning. Abdominal CT scans show the distal stomach and duodeno-jejunostomy to be adherent to the left lobe of the liver (Figure a, oral contrast) by a bridge of tissue which enhances with intravenous contrast suggesting that it is vascular (Figure b, dynamic scan).

perinatal umbilical vein catheterisation although the trauma or sepsis related to the surgery for duodenal atresia and a congenital anomaly of the portal vein[5,6] are other possibilities. Although these are well recognised causes of extrahepatic portal vein thrombosis the latter has not been described in association with duodenal atresia[7].

Extrahepatic portal vein thrombosis produces an increased pressure within the vessels distal to the obstruction while the portal pressure central to the obstruction remains normal[4]. In the present case, however, the portal pressure was only minimally elevated despite occlusion of the previous mesocaval shunt, suggesting that a collateral circulation had developed which was adequate to prevent portal hypertension. Bleeding may therefore have been initiated by the erosion of the mucosa overlying the varices rather than rupture of the varicosities secondary to portal hypertension. This information on portal pressure was crucial to patient management in the present case and emphasises the importance of this measurement in patients with extrahepatic portal vein thrombosis. The conventional management of varices by portal decompression by shunting may not have been appropriate in this case since significant portal hypertension was not present. Furthermore a porto-systemic shunt may have led to reduced liver function and encaphalopathy as a result of deprivation of the liver portal inflow although there is debate on the incidence and severity of this complication following shunts for extrahepatic portal vein thrombosis[8]. The surgical approach which was chosen was therefore to resect the segment of bowel containing the bleeding site and form a Roux en Y jejunal loop. A long term follow up will obviously be required to assess whether resecting an area of the collateral circulation will result in a raised portal pressure and further bleeding or whether varices will re-form at the new anastomosis site.

### *References*

1. Belli, L., Romani, F., Riolo, F., Rondinara, G., Aseni, P., Di Stefano, M., Contorni, L. and Bini, M. (1989) Thrombosis of portal vein in absence of hepatic disease. *Surg. Gyn. Obst.*, **169**, 46–49
2. Rosen, H., Silen, W. and Simon, M. (1967) Selective portal hypertension with isolated duodenojejunal varices. *New Engl. J. Med.*, **277**(22), 1188–1190
3. Bateson, E.M. (1969) Duodenal and antral varices. *Br. J. Radiol.*, **42**, 744–747
4. Itzchak, Y. and Glickman, M. (1977) Duodenal varices in extrahepatic portal obstruction. *Radiology*, **124**, 619–624
5. Webb, L.J. and Sherlock, S. (1979) The Aetiology, Presentation and Natural History of Extra-hepatic Portal Venous Obstruction. *Q. J. Med.*, **48**, 627–639
6. Brown, K., Kaplan, M. and Donowitz, M. (1985) Extrahepatic Portal Venous Thrombosis : Frequent Recognition of Associated Diseases. *J. Clin. Gastroent.*, 7(2), 153–159
7. Odievre, M., Pige, G. and Alagille, D. (1977) Congenital abnormalities associated with extrahepatic portal hypertension. *Arch. Dis. Child*, **52**, 383–385
8. Warren, W.D., Henderson, J.M., Millikan, W.J., Galambos, J.T. and Bryan, F.C. (1988) Management of Variceal Bleeding in Patients with noncirrhotic Portal Vein Thrombosis. *Ann. Surg.*, **207**, 623–634

(Accepted by S.Bengmark on 7 August 1991)

### INVITED COMMENTARY

This interesting case report by Varsamidakis and co-authors challenges several well established principles regarding portal hypertension and variceal hemorrhage: (1)

development of collaterals returns portal pressure toward, but never to, normal; (2) only varices carrying blood away from the liver (hepatofugal collaterals) bleed; and (3) a portal pressure of 12mmHg is necessary for variceal hemorrhage to occur. The patient presented bled from anastomotic varices on several occasions, as documented by endoscopic examination. Portal pressure was measured both indirectly by splenic puncture (13cm of water) and directly by cannulation of a mesenteric vein at surgery (15cm of water). On both occasions, the pressure was well within normal limits. Thus, despite portal vein thrombosis leading to presumably an initial elevation of portal pressure and subsequent collateralization, at the time of their evaluation the pressure had returned to normal. Therefore, the mechanism of variceal bleeding was most likely erosion into a high flow, low pressure varix rather than spontaneous rupture secondary to elevated pressure.

Extrahepatic portal hypertension almost always leads to hepatopetal (towards the liver) as well as hepatofugal (away from the liver) collaterals because the hepatic vascular resistance is normal. The development of hepatopetal collaterals is beneficial as this restores hepatic portal perfusion and lessens the likelihood of hepatic failure and/or encephalopathy. The adherence of the duodenal-jejunal anastomosis to the left lobe of the liver in this patient provided a natural bridge through which hepatopetal collaterals could develop. This resulted in luminal varices, in contrast to the more common situation in which hepatopetal collaterals are most prominent along the hepatoduodenal ligament, and in that location, cannot cause gastrointestinal hemorrhage.

It cannot be determined from this report whether the major site of hepatopetal collateralization, which was resected, was responsible for the normal portal pressure. It would have been desirable for the authors to measure portal pressure after this pathway was interrupted. Likewise, the relatively brief follow-up of this patient prevents a determination as to the long-term effectiveness of the surgical therapy applied.

Layton F. Rikkers
Department of Surgery
University of Nebraska
Medical Centre
Omaha
Nebraska, USA

### INVITED COMMENTARY

The case reported by Varsamidakis and his colleagues nicely points out the many faces of portal hypertension. Although esophageal varices are the commonest cause of gastrointestinal bleeding in this situation, many other sites of variceal bleeding have been observed. Extra-esophageal varices may develop in non-operated patients, particularly those with extrahepatic portal hypertension[1]. However, development of such varices is much favored by postoperative intraperitoneal adhesions[2,3], gastrointestinal anastomoses and ileostomy or colostomy, all these operations can result in venous collaterals between high pressure portal territory and low pressure caval territory. Variceal bleeding has been described in the duodenum[4], small bowel[2,3,5], colon[6], bladder[7], ileal conduit[8], and digestive stoma[9]. The present observation is another example of varices occurring at the site of peritoneal adhesions in a patient with portal vein thrombosis.

An interesting finding of the authors is the low splenic pulp pressure in a patient with variceal bleeding. It is generally acknowledged that the development of collateral veins is not sufficient to result in lowering of portal pressure and prevention of bleeding from varices. Although the present observation is relevant, measurement of superior mesenteric vein and inferior vena caval pressures and of mesocaval pressure gradient would have been preferable in order to assess the presence or the absence of portal hypertension and to confirm that bleeding may occur despite a low portal venous system pressure.

Portal systemic shunts are usually more effective in relieving variceal bleeding than a direct attack on the varices[10]. They have been successfully performed in several cases of extra-esophageal variceal bleeding[4,5,9,11]. Resection of varices may result in more intra peritoneal adhesions and rebleeding[11]. It is now well acknowledged that chronic encephalopathy does not occur in patients with extrahepatic portal hypertension following portal systemic shunts[12]. In the case reported above, the superior mesenteric vein was patent. A mesocaval shunt might have been more appropriate than resection of jejunum in order to avoid long-term recurrent variceal bleeding.

### *References*

1. Bruet, A., Fingerhut, A., Eugene, C. and Fendler, J.P. (1984) Varices intestinales et hypertension portale. *Gastroenterol.Clin.Biol.*, **8**, 725–732
2. Moncure, A.C., Waltman, A.C., Vandersalm, T.J., Linton, R.R., Levine, F.H. and Abbott, W.M. (1976) Gastrointestinal hemorrhage from adhesion-related mesenteric varices. *Am. Surg.*, **183**, 24–29
3. Attias, E., Smadja, C., Vons, C., Trayno, O. and Franco, D. (1987) Bleeding from intestinal varices after a Woman operation. *J. Clin. Gastroenterol.*, **9**, 585–587
4. Shearburn, E.W. and Cooper, D.R. (1966) Duodenal varices treated by portacaval shunt. *Arch. Surg.*, **93**, 425–427
5. Wilson, S.E., Stone, R.T., Christie, J.P. and Passaro, E. (1979) Massive lower gastrointestinal bleeding from intestinal varices. *Arch. Surg.*, **114**, 1158–116
6. Foutch, P.G. and Sivak, M.V. (1984) Colonic variceal hemorrhage after endoscopic injection sclerosis of esophageal varices : a report of three cases. *Am. J. Gastroenterol.*, **79**, 756–760
7. Bruet, A., Fingerhut, A., Lopez, Y., Bergue, A., Taugourdeau, P., Mathe, C., Hillion, D. and Fendler, J.P. (1963) Ileal varices revealed by recurrent hematuria in a patient with portal hypertension and Mekong schistosiomasis. *Am. J. Gastroenterol.*, **78**, 346–350
8. Crooks, K.K., Hensle, T.W., Heney, N.M., Waltman, A. and Irvin, R.J. (1978) Ileal conduit hemorrhage secondary to portal hypertension. *Urology*, **12**, 689–693
9. Adson, M.A. and Fulton, R.E. (1977) The ileal stoma and portal hypertension. An uncommon site of bleeding. *Arch. Surg.*, **112**, 501–504
10. Franco, D. and Smadja, C. (1985) Prevention of recurrent variceal bleeding : surgical procedures. *Clin. Gastroenterol.*, **14**, 233–257
11. Fee, H.J., Taylor, J.B. and O'Connel, T.X. (1977) Bleeding intestinal varices associated with portal hypertension and previous abdominal surgery. *Am. Surg.*, **43**, 760–762
12. Bismuth, H., Franco, D. and Alagille, D. (1980) Portal diversion for portal hypertension in children : the first 90 cases. *Ann. Surg.*, **192**, 18–24

D. Franco

Professor of Digestive Survery

Université de Paris XI

Hôpital Antoine Béclère

Clamart

Paris

